# Sexual and reproductive health interventions for mobile adolescents and young people in sub-Saharan Africa: a scoping review

**DOI:** 10.1186/s12889-025-25119-4

**Published:** 2025-11-11

**Authors:** Langelihle Mlotshwa, Simóne Plüg, Melvin Simuyaba, Natsayi Chimbindi, Darshini Govindasamy, Musonda Simwinga, Tolib Mirzoev, Janet Seeley, Nurture4Youth project team, Virginia Bond, Cora Dee, Nothando Ngwenya, Maryam Shahmanesh, Nondumiso Dlamini, Makhosazane Ntombela, Sinethemba Mabuyakhulu, Siphumelele Ndlovu, Steve Belemu

**Affiliations:** 1 African Centre for Migration and Society, University of the Witwatersrand, Johannesburg, South Africa; 2 Africa Health Research Institute, Durban, KwaZulu-Natal South Africa; 3 Zambart, Lusaka, Zambia; 4 Health Systems Research Unit, South African Medical Research Council, Cape Town, South Africa; 5 London School of Hygiene & Tropical Medicine, London, UK; 6 University College London, London, UK; 7 University of KwaZulu-Natal, Durban, KwaZulu-Natal South Africa

**Keywords:** Interventions, Sexual and reproductive health, Mobility, Migrants, Adolescents, Young people, Sub-Saharan Africa

## Abstract

**Background:**

Mobility among adolescents and young people (AYP) is a key factor influencing their access to sexual and reproductive health rights (SRHR) services. Young migrants are more likely to experience gender-based violence and their access to SRHR services and education is often limited in their new communities. We conducted a scoping review which aimed to map what is known about the extent and type of interventions focused on SRHR for mobile adolescents and young people (mAYP) in sub-Saharan Africa.

**Methods:**

We followed Arksey and O’Mally’s (2005) steps for conducting scoping reviews: 1. Identifying the research question; 2. Identifying relevant studies; 3. Study selection; 4. Charting the data and 5. Collating, summarising and reporting the results. We searched four databases identifying 1,069 articles. After screening, 25 articles were included.

**Results:**

Many studies were conducted in Uganda (n = 12), reported on conflict-related mobility (n = 15), and focused on HIV (n = 11). Two main intervention types were identified: *Links to service provision* and *Knowledge, information, and skills development.* Implementation facilitators included recruiting community health promoters, mentors and peer supporters. Implementation barriers included limited literacy, social norms, access to facilities and stigma associated with accessing services.

**Conclusions:**

Research exploring SRHR interventions for mAYP is limited in sub-Saharan Africa. Future research and interventions should be underpinned by an understanding of young people’s health and wellbeing more broadly, and foreground the social, cultural, religious and economic factors shaping mAYP’s SRH needs.

## Introduction

In sub–Saharan Africa (SSA), adolescents and young people (AYP) aged 15–24 years encounter significant challenges in accessing sexual and reproductive health (SRH) services [1]. AYP in the region have unmet contraceptive needs, contributing to substantial unintended pregnancies, maternal deaths and unsafe abortions [2]. Between 2015 and 2019, an average of 121 million unintended pregnancies were recorded each year, accounting for 48% of all pregnancies [3]. In addition, low and middle income countries accounted for 98% of all unsafe abortions with 41% of this total being young women aged 15–25 years [4]. Further, between 2010 and 2022 adolescent girls and young women aged 15–24 years accounted for 27% of new HIV infections and were three times as likely to acquire HIV as their male counterparts [5]. At the same time, however, Baisley et al. [6] highlight that “young men are twice as likely to die of HIV-related causes” (p. 2) as young women. Melesse et al. [7] observe that most countries in SSA have not made significant progress in meeting the targets set for the 2030 Sustainable Development Goal (SDG) relating to universal access to SRH services, and their legal and policy frameworks do not always adequately support or ensure young people’s access to SRH services. They go on to say that multiple individual, health system, socio-economic and cultural factors contribute to AYP’s limited access to SRH services.

On an individual level, barriers to SRH services for young people in SSA include lack of knowledge about SRH risks and limited information about services available, negative attitudes towards, and misconceptions of, SRH services and practitioners, low self-esteem and self-efficacy and fear of judgement by friends and family [1, 8]. Economically, where free SRH services are not available, or are unsatisfactory, young people are often unable to afford paid SRH services [9]. Misconceptions about contraceptives are also widespread and include the beliefs that they cause infertility, low sex drive and cancer [9]. Cultural and religious beliefs in many SSA countries inhibit sexual education and condom or contraceptive use among young people [10] as they are not considered to be ‘age appropriate’, particularly for young people below the age of 18 years [9, 11]. Further, condom use for preventing HIV/STIs is shaped by complex gender roles and cultural norms within sexual relationships and this largely impacts both partners ability to communicate openly and negotiate protective measures [7]. Inadequate parental support, care and guidance amongst families are also reported to contribute to adverse SRH outcomes and low uptake of SRH services amongst young people in SSA [12]. Young people’s access to SRH services is further hampered by inappropriate service delivery, specifically when health care workers have judgemental attitudes or services lack confidentiality [10, 13].

Mobility among AYP is another key and often overlooked factor influencing young people’s access to SRH services [14, 15]. Livelihood options, education and access to better social services are among the factors that influence mobility among AYP [16, 17]. South Africa has a long-standing history of labour migration rooted in the legacy of colonialism and apartheid [18, 19]. Mobility in present-day South Africa remains high, with the most common pattern involving internal migration from rural or peri-urban areas to urban centres, while maintaining strong connections to places of origin. Mobile adolescents and young people (mAYP) face significant barriers to accessing Sexual and Reproductive Health Rights (SRHR) services, despite legal protections guaranteeing universal health care in South Africa [20]. The challenges include legal status, discrimination, language barriers and lack of familiarity with available services [21].

Research has shown that young migrants are also more likely to experience sexual and gender-based and structural violence [15]. As Vearey [21] highlights, this marginalisation against migrants can manifest in many ways, for example, “in relation to being criminalised by existing policy, poor treatment and violence by the police, and the ways in which ‘othering’ and ‘not-belonging’ result in discrimination and abuse when accessing healthcare” (p. 14). Crankshaw et al. [22] report in their study that young female refugees were at increased risk of transactional sex and unintended pregnancy due to economic vulnerability and service exclusion. Zambia is no different as mAYP continue to face barriers to accessing SRHR, with Zambian youth experiencing the seventh highest incidence of HIV and the eleventh highest fertility rate, worldwide in 2021 and 2020, respectively [23]. The Zambian National Adolescent Health Strategic Plan (2022–2026) [24] emphasises the need to give particular attention to migrant and mobile young people, including those living in refugee camps as a way to improve their SRH*.*

Across SSA, different approaches have been adopted to improve AYP’s access to SRH services. Among the interventions aimed at improving AYP’s access to SRH services is the introduction of youth friendly spaces or services for AYP in health facilities [25, 26], and peer-led and community-based interventions [27–29]. However, AYP (in particular mAYP) in many SSA countries are still not reached by the services offered by these interventions [28, 29].

Several reviews explore access to SRHR in SSA [25, 30–32], commonly exploring the key challenges experienced, particularly for adolescents. These published reviews tend to involve young people broadly, without a specific focus on mobile youth [1, 31, 33, 34] or target internally displaced or refugee women and children – a very particular form of mobility [32, 35, 36]. In this scoping review we aim to bridge this knowledge gap by mapping what is known about the extent and type of SRHR interventions for mAYP in SSA. We aim to contribute to the discourse on key facilitators and barriers to implementation of SRHR interventions amongst mAYP and provide direction for further interventions that will address the challenges faced by mAYP in accessing SRH services in South Africa and Zambia.

## Methodology

We conducted a scoping review to identify and synthesise evidence on SRHR interventions for mAYP, aged 15–35 years, in SSA. This approach was appropriate for reviewing a diverse body of knowledge to explore a broad range of literature, drawing on a range of study designs, with the aim of mapping, synthesising and disseminating what has been done in the area, and identifying the aspects that have not yet been fully explored [37, 38]. We followed the five-stage methodological framework of Arksey and O'Malley [37] for scoping reviews, as outlined below. While these authors provide a clear framework for conducting a scoping review, they provide little detail on how to use this framework in practice [39]. As a result, we also drew on Levac et al.'s [38] recommendations for using their approach, and the JBI guidelines for conducting scoping reviews.

### Stage 1: identifying the research questions

We used the population, concept and context (PCC) framework to guide our research questions, which focused on mAYP (population), SRHR interventions (concept) and SSA countries (context). Our review questions were:

1. What SRHR interventions for mAYP have been implemented in SSA and what extent and form did they take?

2. What factors impacted the implementation and effectiveness of these interventions?

3. What are the wider lessons for co-designing SRHR interventions for mAYP in SSA?

### Stage 2: identifying relevant studies—search strategy

We began by conducting a preliminary search of PubMed and ProQuest databases to gain an overview of the existing body of knowledge, define our key concepts (namely SRHR, interventions, mobile, young people and SSA) and develop and refine key search terms (See Table 1 below). SRHR incorporates a vast range of intersecting categories. Based on the World Health Organization “Framework for operationalizing sexual health and its linkages to reproductive health” [40], we limited the scope for this review and specifically focused on the following: *Prevention and control of HIV and other sexually transmissible infections; Contraception counselling and provision; Sexual function and psychosexual counselling and education; Safe abortion care; and Gender-based violence prevention, support and care* (p. 5). Sexual and reproductive health interventions are understood as actions that are put in place to improve the SRHR of populations by preventing disease or curing or reducing the intensity of an existing disease [41]. The specific focus of this review is mAYP aged 15–35 years [42]. We selected this wide age range as it is the most common age period in which people tend to move location [43, 44] and it is also the age at which people are especially vulnerable to adverse SRH outcomes [45, 46]. When framing mobility, we drew on the International Organization for Migration [47] definition which states that a migrant is “a person who moves away from his or her place of usual residence, whether within a country or across an international border, temporarily or permanently, and for a variety of reasons” (p. 132). We chose the timeframe (2005 onwards) as this was a period of ongoing change in the policy climate in SSA and increased awareness of SRH care as a fundamental human right for all [48].

**Table 1:** Key search terms

Concept	Search words
Sexual and reproductive health	Sexual and reproductive health, sexual health, reproductive health, HIV, PrEP, PEP, Antiretroviral*, ARV, sexually transmitted infection, STI, contraception, contraceptive, condom use, pregnancy, family planning, safe sex, sexual education, abortion, gender-based violence, sexual violence
Young people	Young people, adolescen*, young person, young adult*, young women, young men, teen*, youth
Mobile	Mobil*, migrant, immigrant, refugee, forcibly removed, transient, displaced
Sub-Saharan Africa	Sub-Saharan Africa, Angola, Benin, Botswana, Burkina Faso, Burundi, Cabo Verde, Cameroon, Central African Republic, Chad, Comoros, Congo, Côte d’Ivoire, Democratic Republic of Congo, Djibouti, Equatorial Guinea, Eritrea, Eswatini, Ethiopia, Gabon, Gambia, Ghana, Guinea, Guinea-Bissau, Kenya, Lesotho, Liberia, Madagascar, Malawi, Mali, Mauritania, Mauritius, Mozambique, Namibia, Niger, Nigeria, Rwanda, Sao Tome and Principe, Senegal, Seychelles, Sierra Leone, Somalia, South Africa, South Sudan, Sudan, Tanzania, Togo, Uganda, Zambia, Zimbabwe

Using the terms listed in Table 1 we searched the following four databases: ProQuest, Scopus, PubMed, and Africa-Wide Information. We chose these as they cover the widest range of relevant peer-reviewed literature on the topic. We manually searched the reference lists of relevant articles to identify additional papers for consideration. This yielded a total of 1,644 citations. We exported all references into the literature review software, Rayyan [49] which we used to identify and remove 575 duplicates, resulting in a total of 1,069 citations.

### Stage 3: selecting relevant articles

**Fig. 1:**
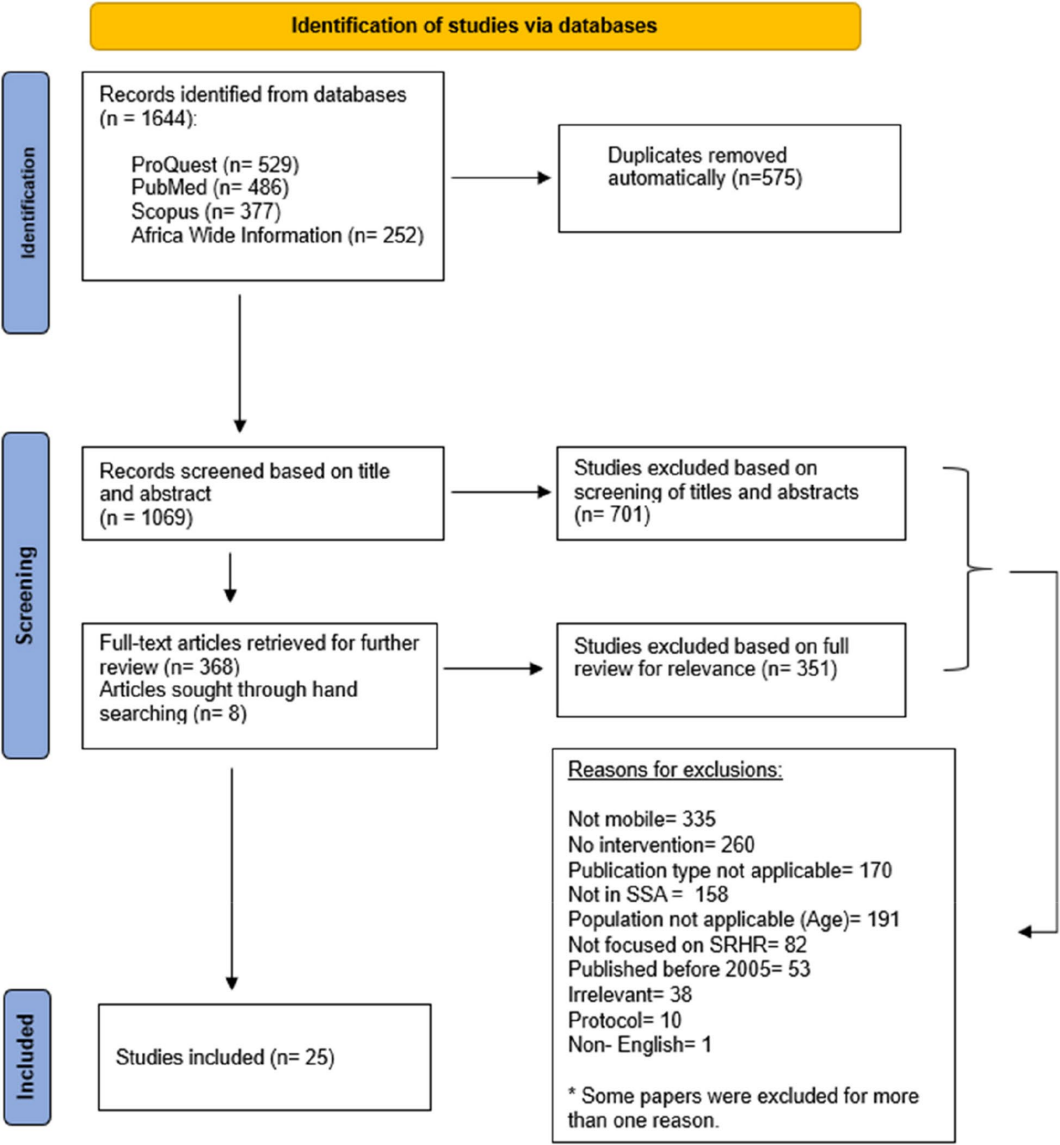
PRISMA flowchart

Three reviewers (MS, LM and SP) independently screened the titles and abstracts of 1,069 identified citations. Again, using Rayyan [49], the reviewers recorded reasons for inclusion or exclusion, and removed any further duplicates that had not been automatically detected. After this stage, we excluded 701 articles and added a further 8 articles [17, 50–56] through manually searching citation lists. The PRISMA Flowchart can be found in Fig. 1 below. Thereafter, we located the full texts of papers, and two reviewers reviewed each paper for relevance.

We applied the inclusion and exclusion criteria, outlined in Table 2 below, and recorded reasons for exclusions. Where full texts were not available online through databases accessible to the reviewers, we approached a university librarian to assist with sourcing the relevant texts. We successfully sourced all articles. It was important to be as inclusive as possible given the limited evidence currently available on SRHR interventions for mAYP in SSA. In this regard, we included peer-reviewed original research studies with no restriction on the study designs and a broad range of SRHR concerns and intervention types.

**Table 2:** Exclusion and inclusion criteria

Inclusion criteria	Description	Exclusion criteria	Description
Topic	Articles exploring young (15–35 years) mobile people and Sexual and Reproductive Health Rights Interventions	Topic	Studies not focused on both young (15–35 years) AND mobile people Studies focused on other (non SRHR) health-related concerns/interventions Studies with no focus on intervention
Context	Studies conducted in any country in Sub-Saharan Africa, including comparative studies with SSA and non-SSA contexts	Context	Studies conducted outside of Sub-Saharan Africa, or comparative studies with no SSA countries
Language	English	Language	Other languages
Publication date	2005 - 2024	Publication date	Before 2005 or after 2024
Publication type	Original Research/Studies	Publication type	Policy documents, grey literature
Type of study	All qualitative, quantitative and mixed method study designs	Type of study	Reviews

When conflicts arose about whether to include articles, a third reviewer reviewed the article, and the decision was discussed amongst all three reviewers. Reviewers also sought out assistance from three further members of the team (DG, TM and JS) where adjudication was required. After the completion of the full text screening, we identified 25 articles for extraction and analysis.

### Stage 4: charting the data

We extracted the following information into Microsoft Excel from each selected publication: study characteristics (citation, year, location, study design, theoretical framework, sample description (including age and sex), type of mobility and SRHR focus area) and intervention characteristics (name, type, components, community or participant involvement, implementation challenges/facilitators, and reported intervention outcomes). Each article had one team member extract the data; and a second team member check their process. The three core team members (MS, LM and SP) worked on this process and discussed any arising issues in weekly team meetings.

### Stage 5: collating, summarising and reporting the results

We collated and analysed the data extracted from the selected studies, and discussed them in two ways. Firstly, we used descriptive statistics to provide an overview of the characteristics of the studies presented in the review as a whole to provide a summary of the extent and type of research conducted in this area. Secondly, a thematic analysis approach [57] was used to explore the key contributions identified across the studies and discuss the implications that these learnings have for future research and interventions for this population. Initial themes were identified from the review questions, and further themes were added from the reviewed articles.

## Results

We start with an overview of the characteristics of all the included papers (Table 3). We then discuss the main intervention approaches, key implementation facilitators and barriers, and overall learnings.

**Table 3:** Summary of study characteristics

Citation	Research method	Study location	Age category	Sex	Type of mobility	SRHR focus area	Intervention type
Abdelmoneium [58]	Qualitative	Sudan	Adolescence	All	Conflict-related	Broad coverage of SRH	Knowledge, information, and skills development; Link to service provision
Compaoré et al. [59]	Qualitative	Burkina Faso	Young people	Female	International and internal migration	Female Nutrition	Link to service provision
Baisley et al. [6]	Quantitative	South Africa	Young people	Male	Internal	HIV	Link to service provision
Bakesiima et al. [60]	Quantitative	Uganda	Adolescence	Female	Conflict-related	Contraception	Knowledge, information, and skills development; Link to service provision
Bakesiima et al. [61]	Quantitative	Uganda	Adolescence	Female	Conflict-related	Contraception	Knowledge, information, and skills development; Link to service provision
Bol et al. [62]	Quantitative	Ethiopia	Adolescence	Female	Conflict-related	Pregnancy	Link to service provision
Braitstein et al. [63]	Quantitative	Kenya	Adolescence and young people	All	Street dwelling	HIV	Link to service provision
Engidaw and Gebremariam [64]	Quantitative	Somalia	Adolescence	Female	Conflict-related	Female Nutrition	Link to service provision
Erulkar et al. [65]	Quantitative	Ethiopia	Adolescence	Female	Internal	HIV	Knowledge, information, and skills development
Jani et al. [66]	Quantitative	Ethiopia	Adolescence	All	Labour-related	HIV	Knowledge, information, and skills development
Kenyon et al. [67]	Quantitative	South Africa	Young people	All	Labour-related	STIs	Link to service provision
Logie et al. [68]	Qualitative	Uganda	Young people	All	Conflict-related	Sexual and gender-based violence	Knowledge, information, and skills development
Logie et al. [54]	Mixed Methods	Uganda	Young people	All	Conflict-related	HIV	Knowledge, information, and skills development; Link to service provision
Logie et al. [69]	Quantitative	Uganda	Young people	All	Conflict-related	HIV	Link to service provision
Logie et al. [70]	Mixed Methods	Uganda	Young people	All	Conflict-related	Sexual and gender-based violence	Knowledge, information, and skills development
Logie et al. [71]	Qualitative	Uganda	Young people	All	Conflict-related	Sexual and gender-based violence	Knowledge, information, and skills development
Logie et al. [72]	Mixed Methods	Uganda	Young people	All	Conflict-related	Sexual and gender-based violence	Knowledge, information, and skills development
Palattiyil et al. [73]	Qualitative	Uganda	Adolescence	All	Conflict-related	Broad coverage of SRH	Knowledge, information, and skills development; Link to service provision
Patel et al. [74]	Quantitative	Uganda	Young people	All	Conflict-related	HIV	Link to service provision
Patel et al. [75]	Quantitative	Uganda	Young people	All	Conflict-related	HIV	Link to service provision
Stark et al. [76]	Quantitative	Ethiopia	Adolescence	Female	Conflict-related	Sexual and gender-based violence	Knowledge, information, and skills development
Temin et al. [77]	Qualitative	South Africa	Adolescence	Female	International migration	HIV	Knowledge, information, and skills development
Townsend et al. [78]	Quantitative	South Africa	Young people	Female	International migration	HIV	Link to service provision
Tumwesige et al. [17]	Qualitative	Uganda	Young people	All	Labour-related	Broad coverage of SRH	Knowledge, information, and skills development; Link to service provision
Wyss et al. [79]	Mixed Methods	Ghana	Young people	All	Internal mobility	HIV	Link to service provision; Knowledge, information, and skills development

### Study characteristics

Our review found that there has been a slight increase in publications focussing on SRHR interventions amongst mAYP over the timeframe selected. In terms of geographical location, the studies spanned eight countries, with most conducted in Uganda (n = 12) followed by South Africa (n = 4) and Ethiopia (n = 4).

From 25 papers, 10 targeted adolescents (± 10–19 years) and 15 targeted young people (± 15–35 years), meaning there are some overlaps between age groups of adolescents and young people. In terms of sex, most of the 25 included articles (n = 15) focused on all individuals, with nine on females alone and only one focused solely on males. With regards to SRHR focus areas, many articles focused on HIV (n = 11). While this is consistent with the severity and urgency of the HIV pandemic in SSA [77], it does highlight a lack of research that explores other important SRH needs such as other STIs (n = 1), pregnancy (n = 1), contraception (n = 2), and more emergent areas such as human papillomavirus and cervical cancer awareness which were not covered amongst this age group in the papers reviewed. It is worth noting that four out of five papers that focused on sexual and gender-based violence were based on one large multi-component study [68, 70–72], inflating the perceived interest in this SRHR focus area.

The 25 included articles also reported on different types of mobility. Most (n = 15) reported on conflict-related mobility (Internally displaced persons and/or Refugees). This focus corresponds with the large proportion of articles located in Uganda. As political conflict in East Africa has been frequent [74], many refugees have sought safety and security in Uganda which has “immigrant-friendly" policies, which offer rights to education, work, healthcare and the right to start a business [73]. Second to this, were articles reporting on labour-related mobility and internal migration (each with n = 3). Fewer articles referred to international migrants (n = 2) or reported on both international and internal migration (n = 1).

Many studies employed a quantitative research methodology (n = 14), most commonly a cross-sectional or survey design (n = 8). Fewer articles drew on qualitative (n = 7) and mixed methods approaches (n = 4). The intervention types represented two broad categories: those that predominantly enabled *Links to service provision* (n = 10) and those that focused mainly on *Knowledge, information, and skills development* (n = 8). These were not mutually exclusive groupings, and many interventions included elements of each, with seven papers discussing interventions that fell equally into both categories. In addition, several interventions (n = 7) across these categories discussed *Mentorship and peer support* as a key component of the intervention.

### Intervention types and implementation

This section presents a more in-depth discussion of the 25 papers, particularly focusing on the intervention components, implementation facilitators and barriers and their implications for future SRHR interventions and research with this population.

#### Links to service provision

The first intervention type identified in this review was *Links to service provision* where interventions either provided health services directly to participants or connected them with further care beyond the study. The main service provided was testing for various SRHR related issues or illnesses. Several articles focused on surveillance and screening for HIV to provide population-based estimates of HIV prevalence, incidence and correlates among mobile young people in different settings [6, 63, 74, 75, 78]. Kenyon et al. [67] described an intervention through which the implementing team collected urine and blood samples to test for herpes simplex virus 2 among young people in South Africa and assessed the risk factors associated with infection among these young people. Other interventions used community-based testing to assess the prevalence and risk factors of pregnancy among adolescent girls [62] and stunting and thinness among refugee adolescent girls in Ethiopia [64]. In these studies, pregnancy was determined using human chorionic gonadotropin pregnancy tests and stunting and thinness through physical measurement of height and weight.

Four articles focused on HIV counselling and testing services [54, 69, 73, 79]. These interventions used different approaches to offer this service. Wyss et al. [79] provided voluntary HIV testing, pre and post-test counselling, referral for HIV care and treatment services including to peer support groups at a facility near a market in temporary tents set up to ensure clients’ privacy. Two articles explored delivery and use of HIV self-testing (HIVST) supported by mHealth (weekly short message service (SMS) check-in using a web-based SMS platform) [69], and HIVST alongside edutainment comics [54] and linkage to confirmatory HIV testing and care among refugee youth (aged 16–24 years).

Other *Links to service provision* interventions focused on the provision of prevention or treatment [54, 58, 59, 61, 69, 79]. Compaoré et al. [59], for example, discussed the provision of iron and folic acid supplements to non-pregnant adolescents and young women aged 15–24 years through home delivery by fieldworkers to improve iron stores of young women before their first pregnancy. Bakesiima et al. [61] reported on an intervention that provided oral contraceptives, injectables and insertion and removal of implants and intra-uterine devices to female refugee adolescents aged 15–19 years. In addition to provision of contraceptives, peer counsellors followed up with adolescent girls and young women who missed appointments or were considered lost to follow up. Abdelmoneium [58] described an intervention focused on prevention and early treatment of disease to lactating and pregnant women by home visits for refugees in a camp in Sudan. While Palattiyil et al. [73] described an intervention component that offered HIV testing, the study also included other intervention components including provision of material supplies (food and SRH products including sanitary pads (for girls) and plastic razors (for boys)) and financial support for school fees, rent and treatment. Condom provision was often incorporated in interventions that provided HIV counselling and testing and provision of contraceptives [61, 69, 73, 79].

Although not their main focus, several of the *Knowledge, information, and skills development* interventions also included some aspects of *Link to service provision*. For example, Temin et al. [77] discussed the use of “opportunity visits” (p. 7) offered as part of the education-focused intervention. Here girls went in groups with a mentor to explore local health care clinics together. This demystified and normalised these processes and procedures, encouraging young women in this sample to more readily take up the available services with less fear. Jani et al. [66] also discussed referral for further care at both government-run and nearby onsite clinics, for HIV testing, as an additional component to the main psychosocial counselling focused intervention.

#### Knowledge, information, and skills development

The second intervention type identified in this review was *Knowledge, information, and skills development* where interventions focused on providing education and improving participants’ life skills more broadly. Many of the articles identified, including those that focused predominantly on providing direct services to mAYP, incorporated some element of information and knowledge sharing. These ranged from the use of edutainment comics [54], through mHealth (SMS support) [69], and various forms of SRH-related counselling [61] to community sensitisation and engagement [79]. Of the articles included, eight had interventions that were predominantly education focused. In these interventions, *Knowledge, information, and skills development* were commonly facilitated in two main ways: through the use of small group education sessions or discussions and through one-on-one counselling or individualised peer support.

Several articles indicated that the main intervention component involved the formation of young people groups (often referred to as “Clubs”)—more commonly for girls—where young migrant people were brought together to be introduced to, and to discuss, important information about SRH and other related social issues. For example, Biruh Tesfa [65] was a program focused on out-of-school girls (aged 10–19) living in slum areas in Ethiopia—most of whom were either migrants or mobile for their work as domestic workers or daily labourers. The programme involved age-segmented “Girls’ Groups”, with facilitated discussions of a life skills and HIV curriculum. The discussions covered a comprehensive range of topics from self-esteem and gender dynamics to STIs and HIV to financial literacy [65]. Similarly, Temin et al. [77], as part of the large multi-national Determined, Resilient, Empowered, AIDS-free, Mentored, and Safe (DREAMS) Initiative, also implemented “Girls’ Clubs” with a focus on HIV prevention amongst international migrant young women living in Durban and Johannesburg in South Africa, and Stark et al. [76] aimed to minimise refugee adolescent girls’ exposure to gender-based violence through the Mentorship, Parental Involvement, and Safe Spaces (COMPASS) programme. The Girls’ Club approach tended to involve weekly sessions, facilitated by older women from the same communities, and also tended to provide a combination of directed learning activities guided by a structured curriculum and more informal time where participants could discuss their own ideas and socialise.

The second common way in which *Knowledge, information, and skills development* were enhanced was through the implementation of one-on-one counselling interventions and more informal individual peer support approaches. These interventions could take different forms and varied in SRHR focus, time, and structure. For example, Bakesiima et al. [60] tested the impact of peer (in comparison to health care practitioner) counselling on uptake of modern contraceptives. With this approach, peer counsellors served as “experts”—challenging common myths about contraceptives and providing practical examples of how various forms of contraceptives work. The sessions took a narrow focus, led by the counsellors, and were one-off 20-min sessions. Alternatively, Jani et al. [66] explored a client-centred, psychosocial counselling approach based on problem-solving therapy. Here the participants identified their most significant everyday struggles related to HIV, substance abuse or violence and the counsellors assisted them in developing their own understandings and strategies for coping with these issues. Here sessions spanned over 3 months, used a broad lens exploring multiple HIV-related topics and allowed participants to direct the focus of the sessions. Several studies used more informal one-on-one lay counselling approaches, commonly carried out by peer navigators. For example, in the *Lending a Hand *intervention [17], peer supporters “facilitated access to healthcare services, provided information and counselling services and offered responsive and person-centred” care (p. 1).

##### Developing social assets and promoting empowerment

Although SRHR information provision was an essential component of *Knowledge, information, and skills development* interventions, many interventions also stressed the importance of developing young migrant people’s social assets or capital – “social norms, values, beliefs, trusts, obligations, relationships, networks, friends, memberships, civic engagement, information flows, and institutions that foster cooperation and collective action” [80] (p. 486)—as key protective factors against poor SRH outcomes in this population. The articles in this review highlighted that the value of the clubs, small groups, or counselling was not solely in the provision of knowledge, but also (perhaps more significantly) in the creation of safe spaces and the development of supportive relationships [65, 76, 77].

The two main ways in which these social assets were promoted were through peer support and mentor guidance. Both Tumwesige et al. [17] and Logie et al. [69] found that peer support was an essential component of their respective SRHR interventions highlighting the value of consistent, flexible, nonjudgemental and caring attention from relatable contemporaries. From these interventions, young people seemed to benefit most from having support from both trusted mentors and peers, providing overlapping albeit slightly different functions. Interestingly, peers tended to be more trusted in terms of information and medical advice [60], whereas mentors or older adults—despite often having more experience in healthcare systems—appeared to be more beneficial in terms of emotional support or nurture [77]. For example, Erulkar et al. [65] highlighted the particular benefit of mentors and recruiters being not only from the same communities, but being adults rather than peers. This allowed the mentors to take on the role of “a pseudo-social worker who can advocate on the girl’s behalf” (p. 190). Older mentors tended to have higher social status allowing them to more effectively negotiate with employers or guardians, and also provide caring and attentive adult support to participants, which is often absent for many young and marginalised women. Tumwesige et al. [17] emphasised how peer supporters themselves were sometimes viewed as “parental figures in their lives who listen and genuinely care for them” (p. 11).

A primary aim of the *Knowledge, information, and skills development* interventions was for young mobile people to be “empowered” through interventions, and the curriculum or topics included financial literacy [77] and resisting gendered power relations [65]. These aimed to facilitate a sense of empowerment [17, 72] and also created opportunities for empowerment through co-production of the intervention itself. *Ngutulu Kagwero* [68, 70–72] and *Todurujo na Kadurok* [54] both used a unique participatory comic book mapping approach incorporated into small group discussions. Young refugees worked together with researchers to create a comic book aimed at improving post-rape healthcare by addressing stigma associated with sexual violence and improving knowledge about post exposure treatment options. This approach was particularly interesting because it drew on a creative and interactive design that allowed young mobile people to actively contribute to the creation and implementation of the intervention. Similarly, Tumwesige et al. [17], centred participants’ ideas in the intervention design, implementing a key stakeholder mapping approach to allow space for young migrants themselves to collectively develop an appropriate intervention based on self-identified risk and protection factors.

#### Implementation facilitators

There was a wide range of implementation facilitators across the articles, with interesting overlap between the two intervention types. Compaoré et al. [59] and Wyss et al. [79] reported the benefit of community engagement, including recruiting community health promoters, mentors and peer supporters who encouraged and supported access to services and improved knowledge of available SRH offerings. Further, community-based peer educators helped with setting up of HIV testing facilities (tents) and packing up at the end of the day [79]. The use of paid (renumerated) peer support workers/fieldworkers encouraged and supported access to services including use of mHealth (SMS), improved uptake and retention of participants to different SRH interventions, reduced stigma, enhanced knowledge about HIV and promoted adherence to treatment [59, 61, 72]. Provision of free services and use of incentives [59] also encouraged service utilisation.

Of particular importance in many of the studies was the use of *local* people and places in intervention delivery. Mentors that were familiar with the language, culture and context allowed participants to feel seen and understood [77]. Similarly with service provision, easy (close) access to community halls or gathering spaces was essential. The spaces themselves seemed particularly beneficial in many interventions, and many participants were able to continue to attend health sessions or gather socially beyond the programme duration citing the value of a context where they could be open and communal [65, 76].

Another key implementation facilitator was the use of participant-centred, peer-driven and often creative approaches like role playing, dramas and comic book activities which allowed young people’s needs, experiences and ideas to be brought to the forefront [68, 72, 76]. Academics, community-based collaborators and refugee youth peer researchers supported the development of comic book scenarios in the studies that utilised graphic medicine as an intervention [68, 70–72]. While community promoters, mentors and peer supporters were the main implementation facilitators, government and international organisations' support for programs also provided necessary financial resources for intervention implementation [73].

#### Implementation barriers

Several implementation challenges were identified, including limited literacy skills, lack of gender sensitive interventions, language, culture and stigma [54, 58, 59, 70, 73]. Further, mobility amongst some participants who had to move for work and school and the change of telephone numbers and places of residence often added to these challenges [59, 79]. This sometimes led to the difficulty for an intervention to reach its intended beneficiaries.

As migrants navigate their journeys, males and females experience migration differently, thus highlighting the importance of ensuring that migrant-focused interventions are both gender sensitive and transformative. When these gendered differences are addressed, this means that all migrants are afforded equitable services and resources ultimately promoting their wellbeing. Some authors reported the lack of gender-related interventions, for example Logie et al. [71] highlighted a lack of support or focus for boys experiencing sexual violence. It was clear in that study that boys who were sexually violated would not be believed by the community if they reported any sexual violence [71].

Navigating cultural and language barriers was identified as a key implementation challenge. Wyss et al. [79] revealed that the barrier in communication in multilingual settings when promoting services was a difficulty in reaching intended beneficiaries. Additionally, culture and religion proved to be a challenge as factors such as group influence and holidays such as Ramadan could impact negatively on SRH service access or delivery [73, 79]. Also, young people, especially adolescent girls and women, needed to seek permission to access SRH services from parents/guardians or husbands which was sometimes denied or difficult to negotiate [59, 60].

Finally, facility level barriers to healthcare access for young migrants included limited resources, such as a lack of medication and supplies, challenges in the linkage of patients to care and stigma associated with accessing services [61, 70, 79]. Further, the lack of or poor renumeration for peer support workers and fieldworkers negatively impacted on the quality of service delivery [79]. Logie et al. [70] elaborated on the need for improved confidentiality from those who worked at health centres, as it was clear that in some situations young people did not report violence, because they feared that clinic staff would share their information and the community would judge them.

Overall, the articles in this review identify multiple individual, community or systemic facilitators and barriers that all had a significant impact on the implementation process. Understanding these factors will allow future interventions to navigate these more effectively.

## Discussion

In this review, we aimed to explore what is known about the extent and type of SRHR interventions for mAYP in SSA and discuss the facilitators and barriers to intervention implementation. A total of 25 articles met the inclusion criteria. Many studies were conducted in Uganda, amongst internally displaced/refugee populations, and addressed HIV. Similar to categorisation used by Agyepong et al. [30], interventions identified in our review tended to either focus on *Links to service provision* or *Knowledge, information, and skills development*. In this section we discuss the implications of our findings for both the design of future interventions with this population, and future scholarship in this area.

Our review highlights several critical gaps in the literature, which can provide an agenda for future research. There has, for example, been relatively little research conducted in SSA focusing on SRHR interventions with mobile young people. This supports the findings of Diaz et al. [81] that highlight the need for more research on SRHR and mobile populations, in a more diverse range of SSA countries. Furthermore, little research focused solely on men or LGBTQIA + populations. While this is consistent with the research in SSA highlighting that HIV impacts the genders disproportionately, with girls and women making up 53% of the people living with HIV [82], research also indicates that men are significantly less likely than women to know their HIV status, or to take up HIV treatment [83]. This highlights a need for further gender-sensitive (understanding the nuanced ways in which gendered socialisation and roles shape the everyday lived-experiences of people [84]) and gender-transformative (focused on “societal transformations towards gender equalities” [85] p.2) research targeting young mobile men and LGBTQIA + populations and their distinct SRH needs [33].

Many *Links to service provision* interventions appear to either introduce a new service or signpost to the existing care, with less attention paid to quality of these SRHR services and limited analyses of the responsiveness of wider health systems to the needs of mAYP. The concept of health systems responsiveness comprises multiple domains such as dignity, autonomy, confidentiality, prompt attention, quality of amenities, access to support networks, choice of service provider and trust [86–88], and can represent one possible heuristic to inform in-depth understanding of the underlying theoretical bases and specific mechanisms of SRHR interventions [89] in future research.

Our review shows that there was also a strong focus on HIV, and not broader SRHR concerns, with several studies providing screening for HIV for surveillance purposes [6, 74, 75, 78], with no further intervention components. This mirrors what Embleton et al. [25] argue: that there is a need for a broader understanding of young people’s health and wellbeing, and a particular need for research to go beyond surveillance or screening, and instead draw on “Well-grounded theoretically informed intervention models” [81] (p.436) that address the social, cultural and religious factors [33, 45] connected to mAYP’s SRH needs. Here, foregrounding young mobile people’s own perspectives and encouraging mAYP-led interventions would provide an important addition to this literature.

Our findings also provide some important learnings to consider when designing and implementing future SRHR interventions amongst mAYP. Community engagement, mobilisation and sensitisation (including partner involvement) are key for any successful implementation and acceptability of SRH interventions [59, 61, 79]. These help demystify misconceptions about the interventions, raise awareness, and encourage service uptake. To improve the intervention reach for mobile populations, mapping locations where they are likely to be found or travel to, and directly approaching participants in these areas, is essential [59, 65, 79]. This could also help with tracing mobile populations who may be lost to follow up during intervention implementation. Careful consideration should be taken when choosing where and who provides SRH services for young mobile populations. Spaces offering privacy and safety and located near where mobile populations are found, should be prioritised [79]. Having these safe spaces available to young people on an ongoing basis also meets a significant need in this population [65, 76]. Peer support workers should be involved in (and adequately remunerated for) service provision for mAYP who may experience disrupted social networks [69, 79]. This highlights the need for interventions to build social assets and prioritise participant-centred, consistent and nonjudgemental care through attentive and responsive mentoring [17, 65, 77].

Time is a significant factor to consider in SRHR interventions with mobile youth. Services offered with consideration of times when mAYP are likely to be free, for instance, weekends, around school classes, or after working hours, are likely to yield better outcomes [76, 79]. Interventions that require less frequent interactions with target populations are more likely to be successful, for instance providing long-acting reversible contraceptives (LARC) is more likely to be acceptable compared to providing short-acting reversible contraceptives [61]. This, however, is less feasible with educational or social capital focused approaches, as psychosocial or attitudinal change, relationship building, and societal shifts are slow processes that require sustained engagement [60, 66, 77].

Provision of *free and integrated* (comprehensive) SRH services for mAYP could improve uptake of services [46], reduce stigma and promote linkage to care [59, 79]. For example, providing novel and multipronged approaches (such as edutainment, mHealth, VCT services *and* condoms) will improve knowledge [54, 69, 82], promote prevention, and enhance linkage to care. Socio-cultural and gender differences between mAYP need to be considered when implementing interventions as they might have diverse backgrounds that shape their experiences and impact both their SRH needs and ability to access these services [73]. Here, capacity building for community health workers/promoters, peer educators and health care providers that enhances practice skills, reduces stigma, improves confidentiality and builds acceptance and trust [58, 68, 71, 72], would strengthen the ability of individuals, organisations and systems to more effectively deliver SRHR education, prevention and treatment efforts to mAYP [90].

In addition, gender, culture, and religion focused interventions, that address hegemonic norms, and how these impact relationships and beliefs, remain an important and underexplored area [60]. This reflects Leite et al.'s [33] findings, highlighting the need for more “social norms interventions” which, like gender-transformative interventions, are aimed at “i) changing social expectations, ii) catalysing and reinforcing change, and iii) publicising and diffusing change” (p. 5). As also discussed by Bakesiima et al. [60] and Stark et al. [76], for these programmes to be most effective they need to target a range of groups connected to mAYP, not just mAYP themselves [33]. Linked to this is the need for research in this area to confront the ongoing systematic and structural barriers that perpetuate risks for mAYP [77]. Malhotra et al. [91] emphasise the need for adolescent SRHR interventions to adequately address structural determinants like poverty and gender inequality arguing that “a shift in these more structural and institutional drivers is essential for the desired shift in norms and behaviors around [adolescent SRHR] to be both long term and pervasive” (p. 15).

A summary of our recommendations for future interventions and future research can be found in Table 4.

**Table 4:** Summary of Recommendations

Recommendations for Future Research	Recommendations for Future Interventions
More research on occupation (labour or education) related mobility amongst young people	Interventions should be underpinned by an understanding of young people’s health and wellbeing more broadly
More research on mobile LGBTQIA + and male populations	Interventions should foreground the social, cultural, religious and economic factors shaping mAYP’s SRHR needs
Assessment of the *quality* of SRHR services for mAYP is needed.​	Interventions should prioritise participant-centred, consistent and nonjudgemental care
Further analyses of health system *responsiveness* to mAYP’s needs would be beneficial	Careful consideration of the time, place and means of delivery of SRHR interventions for mAYP is essential
More research on co-development of SRHR interventions with mAYP	Successful implementation and acceptability require meaningful engagement of communities including continuous sensitisation and involvement of key partners and stakeholders

## Study strengths and limitations

In this study we followed a well-established, systematic and clear approach to review the literature and report results, and we sought to minimise bias with a two-stage screening process using two reviewers and adjudicators where conflicts arose. We have, however, identified several limitations of our review. One limitation is that intervention design and implementation are not always well-articulated or fully described in academic, peer-reviewed literature. Inclusion of grey literature may have added to the comprehensiveness of our results. A further limitation was the inclusion of articles published in English only. SSA is highly linguistically diverse and including papers published in other languages would have allowed for a broader discussion of SRHR interventions for mAYP in SSA. In addition, very few papers retrieved from our search focused on our specific age range; most papers found were full population studies. While these articles did often have samples with large numbers of adolescents and/or young people, the interventions in these papers were not designed *specifically for* adolescents or young people and were thus excluded. It is possible that some interventions, designed for full population cohorts, may have relevance for younger populations too.

An additional limitation is that we may not have searched individually for all possible occupations or life circumstances that demand high levels of mobility. For example, long-haul trucking, pastoralism, or sex work (amongst others), all tend to require people to be mobile by the nature of the work. Similarly, broader life circumstances like homelessness or street dwelling may also, by necessity, require high mobility. As a result, articles that focused on these particular occupations or populations may have not mentioned or discussed mobility, as this is assumed to be a well-known or accepted component of the occupation or way of life and were thus “missed” within the searching procedure. This could explain why many of the studies in this review focused on conflict-related mobility, working with refugee or internally displaced populations, with much fewer articles discussing other forms of mobility, such as labour-related mobility. Similarly, not all SRHR components were individually searched for. As discussed in the methodology, we limited the search by choosing not to specifically search for fertility care or antenatal, intrapartum or postnatal care. This explains the lack of articles found in this sample. Finally, conducting an analysis of the quality of studies included, and the cost-effectiveness of interventions discussed, was beyond this scoping review. Future reviews that focus specifically on particular mobile-inclined occupations, on young mobile people’s needs in terms of fertility or antenatal care, and the cost-effectiveness of implemented interventions, are needed.

## Conclusion

Mobility is an important factor that impacts SRHR of young people, particularly in relation to HIV risk and gender-based violence. The articles identified in this review provide useful lessons for implementing intervention-based research amongst this population. However, more scholarship that draws on theoretically-informed, comprehensive interventions to shift the social, cultural, religious and economic factors shaping mAYP’s SRH experiences and needs is still necessary. Multistakeholder collaboration that recognises that matters relating to young migrants and SRHR are complex, extends beyond the boundaries of individual sectors, and provides a coordinated effort for effective solutions, is critical [41]. This review highlights that there is a need for meaningful engagement and cooperative partnerships between sectors, departments and stakeholders (for example education, health, social services and communities) to identify common goals and joint approaches that address these complex matters, collectively [92].

## Data Availability

All data generated or analysed during this study are included in this published article.

## Abbreviations

AYP: Adolescents and young people
HIV: Human Immunodeficiency Virus
HIVST: HIV self-testing
LGBTQIA +: Lesbian, Gay, Bisexual, Transgender, Queer, Questioning, Intersex, and Asexual with the " + " indicating the inclusion of other diverse sexual orientations and gender identities
mAYP: Mobile adolescents and young people
mHealth: Mobile health
PEP: Post-exposure prophylaxis
PrEP: Pre-exposure prophylaxis
SRH: Sexual and reproductive health
SRHR: Sexual and reproductive health rights
SSA: Sub-Saharan Africa
STI: Sexually transmitted infection
